# Transactions of the Southern Gynecological Association

**Published:** 1891-10

**Authors:** 


### Book Reviews.

---

Transactions of the Southern Surgical and Gynecological Association. Vol. III. Published by the Association.

It contains some valuable papers, presenting the advance thought of the day pertaining to surgery and gynecology, deserving careful perusal and study. The character of the articles is such that a much older society might consider itself fortunate to receive such contributions. Although the Southern Surgical and Gynecological Association has but passed its third mile-stone, it numbers as active workers and contributors some of the best talent this country affords.

To enter into the discussion of the merits of the articles presented would consume more space than is allotted to this review. The address of the president regarding the health of the American girl as imperiled by the social conditions of the day should be read by every physician, and when opportunity affords impress on the mother and daughter the signals of danger and advice contained in this article. If such a paper could be placed in every home and its teachings heeded, a vast amount of suffering and disease would be avoided, and its influence soon be made manifest in improving the physical, mental and moral condition of the mothers of future generations. In the list of contributors to this volume we find such names as Engleman, Kelley, Price, Reamy, McMurty, Roberts and others, which are a sufficient guarantee of the worth of this volume. The papers, with their discussions, form a book well worthy a place in a thinking physician's library.

